# Blockchain-enabled EHR access auditing: Enhancing healthcare data security

**DOI:** 10.1016/j.heliyon.2024.e34407

**Published:** 2024-08-10

**Authors:** Faheem Ullah, Jingsha He, Nafei Zhu, Ahsan Wajahat, Ahsan Nazir, Sirajuddin Qureshi, Muhammad Salman Pathan, Soumyabrata Dev

**Affiliations:** a Faculty of Information Technology, Beijing University of Technology, Beijing, China; b School of Computing, Dublin City University, Dublin, Ireland; c School of Computer Science, University College Dublin, Dublin, Ireland

**Keywords:** EHR audit trail, EHR access control policy verification, Smart contract, EHR audit logs, Access control pattern, Purpose-based access control

## Abstract

In the realm of modern healthcare, Electronic Health Records *EHR* serve as invaluable assets, yet they also pose significant security challenges. The absence of *EHR* access auditing mechanisms, which includes the *EHR* audit trails, results in accountability gaps and magnifies security vulnerabilities. This situation effectively paves the way for unauthorized data alterations to occur without detection or consequences. Inadequate *EHR* compliance auditing procedures, particularly in verifying and validating access control policies, expose healthcare organizations to risks such as data breaches, and unauthorized data usage. These vulnerabilities result from unchecked unauthorized access activities. Additionally, the absence of *EHR* audit logs complicates investigations, weakens proactive security measures, and raises concerns to put healthcare institutions at risk. This study addresses the pressing need for robust *EHR* auditing systems designed to scrutinize access to *EHR* data, encompassing who accesses it, when, and for what purpose. Our research delves into the complex field of *EHR* auditing, which includes establishing an immutable audit trail to enhance data security through blockchain technology. We also integrate Purpose-Based Access Control (*PBAC*) alongside smart contracts to strengthen compliance auditing by validating access legitimacy and reducing unauthorized entries. Our contributions encompass the creation of audit trail of *EHR* access, compliance auditing via *PBAC* policy verification, the generation of audit logs, and the derivation of data-driven insights, fortifying *EHR* access security.

## 1 Introduction

*EHR* are essential for improving the quality, safety, and efficiency of healthcare however, they also pose significant risks to the privacy and security of patients' personal and health information [1]. It is essential to know who is accessing *EHR* data, when and for what purpose [2]. Without adequate audit systems to ensure accountability, *EHR* systems remain vulnerable to undetected misuse, both malicious and accidental. Users could modify or delete protected health information without these actions being traceable to the modifier. To protect sensitive *EHR* data, healthcare organizations need to monitor and audit *EHR* access activities regularly and effectively [3].

These include who has access to *EHR* data and their roles and permissions, when, where, how, and for what purpose *EHR* data is accessed, viewed, created, updated, as well as what actions are performed on EHR data. Furthermore, need to track any changes made to *EHR* data e.g. additions, edits, corrections, or deletions and any errors, incidents, or breaches involving *EHR* data e.g. unauthorized access, disclosure etc [4]. It is also necessary to examine whether access control policies for *EHR* data, have been implemented correctly and are functioning as intended. This involves testing the system by attempting to access patient records or information without proper authorization [5].

**Fictional Case Study: Ensuring Security and Efficiency in EHR Management Scenario:** Green Valley Medical Center (fictional name), a state-of-the-art hospital, is known for its excellent patient care and cutting-edge medical technology. However, due to financial constraints and a lack of focus on IT infrastructure upgrades, the hospital has not optimized its *EHR* system's audit trail management. The system suffers from computational inefficiencies, making it difficult to handle large volumes of data. Incident: During a routine system upgrade, an IT staff member inadvertently introduces a vulnerability into the network. An external attacker exploits this vulnerability to gain access to the *EHR* system. Over several months, the attacker systematically alters patient records, modifying diagnoses, prescriptions, and treatment histories. The hospital's outdated audit trail system fails to detect these unauthorized changes promptly due to its inability to process the audit logs efficiently.

**Impact:**

**1. Patient Safety and Data Integrity:** Affected patients begin receiving incorrect treatments based on the altered *EHR*. One patient, John Doe, is misdiagnosed with a condition requiring surgery. The unnecessary procedure leads to complications, resulting in extended hospital stays and additional medical costs. The hospital's reputation for patient safety is severely damaged.

**2. Delayed Detection and Response:** The inefficiency of the audit trail system means it takes the hospital weeks to identify the unauthorized access and the extent of the data tampering. This delay allows the attacker to continue their activities undetected, further compromising patient records. The extended response time exacerbates the harm to patients and erodes trust in the hospital's ability to protect sensitive information.

**3. Regulatory Non-Compliance and Legal Consequences:** Green Valley Medical Center is required to comply with Health Insurance Portability and Accountability Act *(HIPAA)* regulations, which mandate regular compliance audits and the maintenance of detailed audit logs. Due to the inefficiencies in their system, the hospital fails to meet these requirements, resulting in regulatory violations. The hospital faces substantial fines and potential legal actions from affected patients, leading to significant financial losses.

**4. Financial and Reputational Damage:** News of the data breach spreads rapidly, leading to a public outcry. Patients and their families lose trust in the hospital, resulting in a decline in patient admissions. The hospital's financial stability is threatened as it deals with the costs of legal battles, regulatory fines, and efforts to rebuild its reputation. The long-term impact includes a decrease in revenue and potential layoffs of hospital staff.

**5. Performance Limitations and Operational Inefficiencies:** The outdated audit trail system's computational constraints lead to slow processing times, causing delays in accessing patient records during critical moments. Healthcare providers experience frustration as they struggle to retrieve accurate and timely information, impacting their ability to deliver effective care. The hospital's overall efficiency is compromised, affecting its capacity to serve the community effectively. This fictional scenario highlights the importance of addressing computational challenges in managing *EHR* and audit trails. By investing in advanced audit trail management solutions, healthcare organizations can ensure the security and integrity of patient data, comply with regulatory requirements, and maintain operational efficiency. Failure to prioritize these aspects not only risks patient safety and data integrity but also exposes the organization to significant financial and reputational damage. Thus healthcare entities must recognize the value of robust audit trail systems in protecting sensitive information, facilitating timely responses to security incidents, and ensuring seamless access to patient data.

This can be achieved by monitoring and auditing EHR access and activity, which can ensure that following the relevant laws, regulations, standards, and guidelines, such as the *HIPAA*, the Health Information Technology for Economic and Clinical Health *(HITECH)* Act, and the Meaningful Use criteria [6] [7] [8]. Blockchain technology has a transformative impact on *EHR* auditing, audit trail management, compliance audits, and audit logs. It establishes an immutable audit trail, recording every *EHR* access and modification event, ensuring the authenticity of actions within the *EHR* system to prevent unauthorized alterations or deletions of critical patient data. Through cryptographic hashing and decentralized consensus, blockchain enhances data security, making it difficult for malicious actors to tamper with audit logs, thus maintaining the integrity of records during compliance audits [9].

Moreover, blockchain's decentralized nature enables real-time tracking of *EHR* activities, allowing auditors to monitor access, alterations, and data sharing instantly, which in turn facilitates proactive responses to breaches or policy violations. Blockchain also empowers patients by granting them control over their health data through the use of smart contracts. These contracts specify who can access their records and under what conditions, thereby ensuring compliance with privacy regulations [10].

Applying *PBAC* policies ensures access to *EHR* aligns with predefined purposes. It has important role in compliance auditing by verifying access legitimacy and contributing to precise audit trails. With the integration of smart contracts, *PBAC* enhances security and accountability [11].

**Figure 1:**
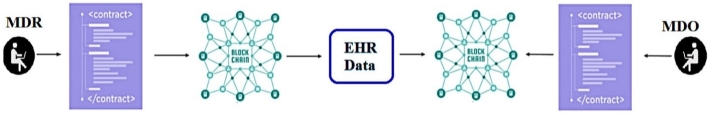
EHR Access with Blockchain.

Smart contracts enable *PBAC* to validate that each access instance adheres to defined purposes, reducing unauthorized entries into the *EHR* system. This combination ensures accurate and granular audit trails, simplifying the detection of irregularities or policy violations for auditors. See Fig. 1.

### 1.1 Research gap and our contribution

In this study, we undertake a comprehensive examination of crucial yet underexplored facets within the *EHR* domain. Specifically, our investigation centers on audit trail, audit logs, access control policy validation for compliance auditing, an area that has thus far received limited scholarly attention. Remarkably, our analysis reveals a notable research gap in the integration of smart contracts and *PBAC* policies mechanisms for *EHR* auditing. To the best of our knowledge, no prior work has ventured into this territory [12], [13], [14] and [15]. As pioneers in this endeavor, we aim to shed light on these critical dimensions, contributing to the enhancement of *EHR* systems by bolstering their security, transparency, and compliance measures. Our contributions encompass the following key aspects:

**Comprehensive Audit Trail:** While audit trails in *EHR* systems have received attention, there is a notable research gap in the development of a comprehensive audit trail that includes detailed user access records, timestamps, and purposes for access. Furthermore, to the best of our knowledge, no prior work has explored the use of smart contracts in *EHR* audit trails. Our research aims to address both of these research gaps, enhancing transparency and security in *EHR* systems.

We introduce an innovative approach of presenting an audit trail of *EHR* access instances by leveraging smart contracts. Through the decentralized nature of blockchain, we establish an immutable audit trail that transparently records transaction details, including sender and recipient information, transaction hashes, gas consumption, and digital signatures. The approach ensures data integrity and enables efficient tracking of details related to *EHR* access, while eliminating the need for intermediaries.

**Access Control Policy Verification for Compliance Auditing:** Although it's widely acknowledged that verifying access control policies for compliance auditing is a important aspect of *EHR* systems, there exists a significant void in the utilization of smart contracts for this specific purpose. Surprisingly, there is no prior research that has ventured into the realm of using smart contracts to conduct compliance auditing within *EHR* systems. Our research is dedicated to closing this gap by introducing an inventive approach to improve the efficiency and precision of compliance auditing in *EHR* systems. Our contribution encompasses the design and implementation of an advanced compliance auditing framework seamlessly integrated into smart contracts, functioning within the context of *PBAC* policies. The system ensures adherence to validation checks, denying unauthorized access attempts, verifying user identities, and fostering transparent transactional interactions within the blockchain ecosystem facilitating compliance auditing processes. Additionally, we provide a dependable mechanism for financial verification, enhancing trust and compliance within decentralized systems during the compliance auditing process.

**Audit Logs**: Our contribution involves generating audit logs derived from the system's audit trail and access control policy verification processes. These logs offer a concise overview of access requests, outcomes, and related details, aiding in the efficient management and monitoring of data access. By documenting both successful and failed access attempts, along with the reasons for any failures, such as invalid purposes or *EHR*, our approach ensures accountability within the system.

**Data-Driven Insights:** To provide a view of *EHR* access patterns, we leverage a dataset. This dataset aids in visualizing various access patterns, offering valuable insights for a better understanding of *EHR* access auditing.

The subsequent sections of this paper provide a comprehensive breakdown of the *EHR* access auditing process. Section 2 offers a concise literature review, summarizing pertinent existing research. In Section 3, we present the system architecture, detailing its components and their interactions. Section 4 is dedicated to result analysis, discussing implications and connections to existing literature and research objectives. Finally, in Section 5, we conclude and outline directions for future work.

## 2 Literature review

Current auditing schemes, often centralized and susceptible to single points of failure, face efficiency challenges, especially when dealing with frequently accessed *EHR* data [16][17]. To enhance accuracy and mitigate these vulnerabilities, Azaria et al. [18] propose real-time and deterministic auditing techniques, reducing storage overhead and improving scalability without relying on blockchain technology.

Blockchain technology, as highlighted by Zheng et al. [19][20], offers decentralization, trustlessness, and tamper-proof capabilities, making it resilient due to its collaborative node architecture. In this context, an approach by [21] Fan et al. called *Dredas* introduces decentralized auditing for *IIOT* data, replacing the need for a trusted third-party auditor with a tamper-resistant smart contract. In a broader context, Ahene et al. [22] integrate Heterogeneous Signcryption with Proxy Re-Encryption *(HSC-PRE)* and blockchain technology to create a secure, accessible, and auditable *EHR* system. Al Baqari et al. propose the integration of biometric-based blockchain technology into *EHR* systems, including Receptionist *IDs* for auditing purposes [23]. In [24], the Jabbar et al. suggested a blockchain-based framework method called *BiiMED*, to manage and validate shared data between medical providers who register health data in the cloud and share *EHR* of patients. Their method presented the Trusted Third Party Auditor *(TTPA)* based on blockchain technology, responsible for endorsing the exchanged data.

Sujihelen et al. explore *EHR* audits within the context of blockchain technology and privacy enhancement using Hyperledger Fabric [25], while Adlam et al. present a permissioned blockchain framework for *EHR* audit trails, incorporating smart contracts [26]. Chelladurai et al. introduce a peer-to-peer blockchain network for secure *EHR* data storage and sharing [27], and Dubovitskaya et al.'s *ACTION-EHR* study focuses on securing sensitive *EHR* data using permissioned blockchain [28]. To incorporate *EHR* auditing into network storage services, Xu et al. introduce an approach that combines client-side deduplication and blockchain technology, streamlining auditing and enhancing data management [2].

To underscore the significance of audit compliance across various domains, following approaches take center stage. In the healthcare sector, an architectural proposal by Roman et al. [29] harnesses blockchain technology and *FHIR* standards to facilitate fine-grained access control, focusing on transparency and *GDPR* compliance in consent management. Shifting focus to the realm of *IoT*, another study by Cha et al. introduces a blockchain-enabled auditing system, aligning seamlessly with *ISO/IEC 15408-2* standards, demonstrating the harmonious integration of *IoT* auditing principles with decentralization and scalability requirements [30] [31].

In practical scenarios, as demonstrated by ALDOSARI and their survey team [32], who conducted the evaluation of 17 different audit functions using an audit vignette in a test domain. Their study is prompted by the growing utilization of *EHR* systems within Saudi hospitals and study endeavors to assess the vital audit capabilities and functions while also determining the compliance level of the *EHR* system deployed at King Abdul-Aziz Medical City hospital in Riyadh, Saudi Arabia.

To further evaluate the applications of *EHR* audit trail logs, Wu et al. [33] used *EHR* audit trail logs for clinical workflow analysis, comparing them to known workflow changes in a healthcare organization. Their proposed scheme assesses three hypotheses related to *EHR* system enhancements, log data manipulation, and behavioral patterns of clinicians. In a related study, Rose et al. [34] aimed to determine if contextual factors derived from *EHR* audit log data can explain variations in clinical process outcomes, focusing on door-to-needle time *(DNT)* for acute ischemic stroke *(AIS)* patients receiving tissue plasminogen activator *(TPA)* in three Northern California health systems.

The Shahook et al. [35] presented survey of blockchain-based access control methods for *EHR*, leveraging smart contracts for secure identification, authentication, and authorization of clients, offer insights into strengthening the security and integrity of *EHR* data. While the primary focus is not be on auditing purpose, the access control mechanisms they propose contribute significantly to creating an auditable environment by ensuring accountability and traceability of data interactions. Likewise, Tith et al. [36] proposes a decentralized system leveraging Hyperledger Fabric blockchain technology to address challenges in accessing and sharing patient records across *EHR* systems without centralized supervision, provides valuable insights and infrastructure that can inform auditing practices within the healthcare sector. See Table 1.

**Table 1:** Comparison of Studies.

Study	Approach & Details	Key Findings/ Contribution	Limitations
[21]	Decentralized auditing scheme for IIOT using Ethereum smart contracts. Supports batch and dynamic auditing.	Ensures strong security of data auditing using nonce, delay function, BLS signature, and bilinear pairing technology. Supports batch and dynamic auditing, paying for auditing and punishing malicious behavior using smart contract deposits. Absence of Audit Trails and Compliance Auditing information	Absence of Audit Trails and Compliance Auditing information
[22]	Introduces HSC-PRE scheme for auditable, EHR systems using blockchain technology.	Proposes HSC-PRE scheme addressing security challenges of EHR systems, integrates with blockchain for auditability and interoperability. Absence of Audit Trails information and Compliance Auditing	Absence of Audit Trails information and Compliance Auditing
[34]	Utilizes EHR audit log data to analyze contextual factors affecting clinical process outcomes.	By utilizing EHR audit log data, finds that greater prior team experience, especially with acute ischemic stroke cases, leads to shorter DNT. Not backbone by Blockchain Absence of Audit Trails and Compliance Auditing information	Absence of Audit Trails and Compliance Auditing information
[24]	Utilized Ethereum blockchain EHR+ IoMT Healthcare data sharing with a decentralized Trusted Third Party Auditor (TTPA).	Using of cloud servers might generate security issues Absence of Audit Trails and Compliance Auditing information	Absence of Audit Trails and Compliance Auditing information
[37]	Utilized Hyperledger blockchain for EHR management, access control and for audit.	Proposed a mechanism to save hashes of stored EHR data and control access when retrieving it helpful for auditing. Lacks detailed discussions on the implementation, experiments, and results regarding EHR auditing, Absence of Audit Trails and Compliance Auditing information	Absence of Audit Trails and Compliance Auditing information

## 3 Framework architecture

**Figure 2:**
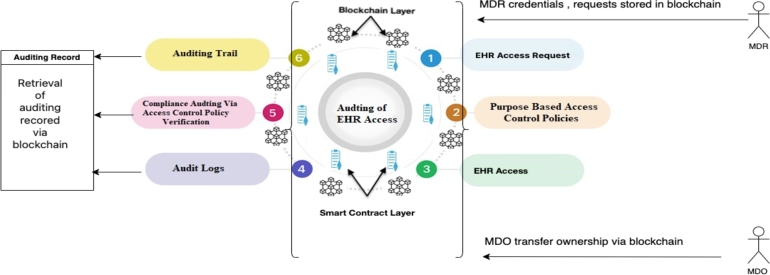
Components and Steps Involved in the Auditing of EHR Access.

**Figure 3:**
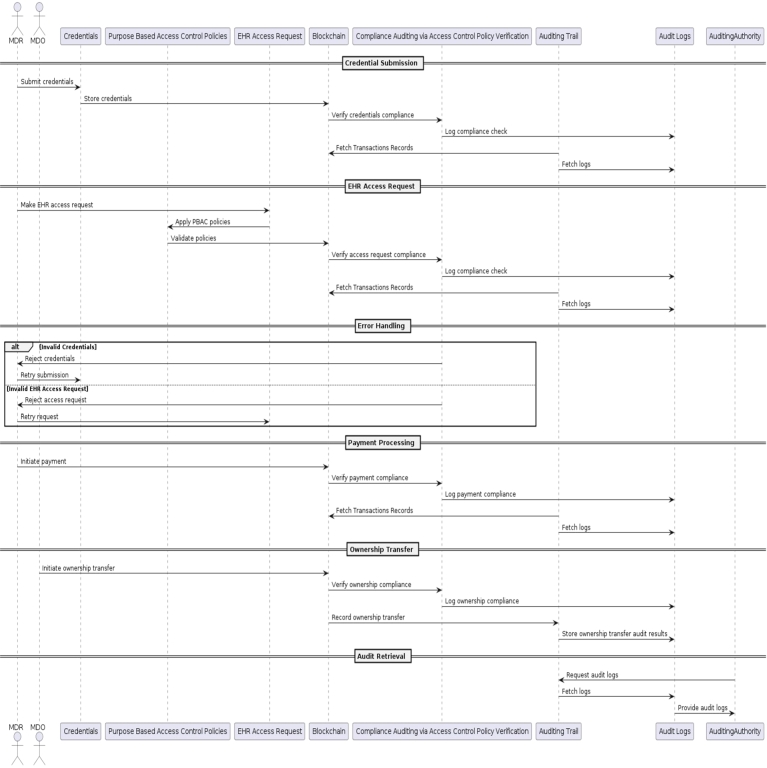
Overview for the Auditing of EHR Access.

Our proposed auditing scheme relies on the integration of the following pivotal components: *EHR* entities (*EHR* data, *MDO*, *MDR*), *PBAC* policies, the implementation of PBAC policies through Smart Contracts, 3 types of auditing namely audit trail, compliance auditing and audit logs as shown in Fig. 2, Fig. 3 and in Fig. 5. These interconnected elements form the foundation of our comprehensive approach to auditing *EHR* access and ensuring *EHR* data security.

### 3.1 Preliminaries

Auditing is a fundamental process that ensures transparency, accountability, and compliance during the granting of access to *EHR*s for research purposes. It involves multiple stakeholders, each playing a role in *EHR* data while facilitating legitimate research efforts. Let's explore the key components in more detail.

#### 3.1.1 EHR stakeholders and data ownership

The *MDO* refers to the patient who possesses and controls access to the *EHR*s, serving as the primary actor. Another important entity is the *MDR*, which could be a researcher or institution seeking access to *EHR*s for research purposes. The *MDO* is responsible for deciding whether to share their medical data and under what circumstances. Before granting access, the *MDO* must be informed about the research purpose and agree to share the *EHR*s via blockchain. To gain access, the *MDR* must provide a clear and specific research purpose, aligning with defined *PBAC* policies. Access is granted or denied based on whether their research purpose aligns with the policy criteria.

#### 3.1.2 PBAC policies for distribution of rights and responsibilities

*PBAC* policies govern the distribution of rights and responsibilities regarding the sharing of *EHR*s for data ownership via blockchain for research purposes. The authorized and qualified *EHR* requesters are granted access for data ownership, contingent upon providing a clear and specific research purpose statement. To preserve data privacy, access is limited to relevant information aligned with predefined authorized research topics, and strict data retention periods are enforced. These policies are verified via compliance auditing, ensuring that policies are rigorously examined and adhere to regulatory requirements, thereby enhancing transparency and accountability in data access management.

#### 3.1.3 Smart contract

Prior user consent from the *MDO* is mandatory and is facilitated by robust privacy measures, including data encryption and anonymization. In this process, smart contracts play an important role in ensuring policy enforcement [38]. A comprehensive audit trail records all access events, and smart contracts guarantee the adherence to these policies, promoting transparency, accountability, and full compliance throughout the entire data sharing process. The auditing process involves three main types of activities.

#### 3.1.4 Audit trail

**Figure 4:**
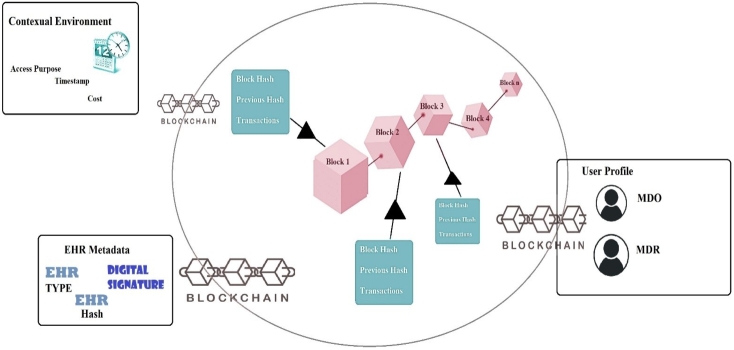
Audit Trail in Blockchain.

The audit trail as shown in Fig. 4 serves as a detailed record of all access events related to *EHR* data. It captures important information, including user profiles *(UP)*, *EHR* metadata *(EM)*, and Transaction Context *(TC)* for each access event. The *UP*function tracks user-related details, such as user identification and access privileges. The EM function records metadata about the *EHR*, such as *EHR* owner or initiator, *EHR*'s digital signature and hash etc. The *TC* component captures contextual information, like access purpose and timestamps, providing a view of each access event.

**Figure 5:**
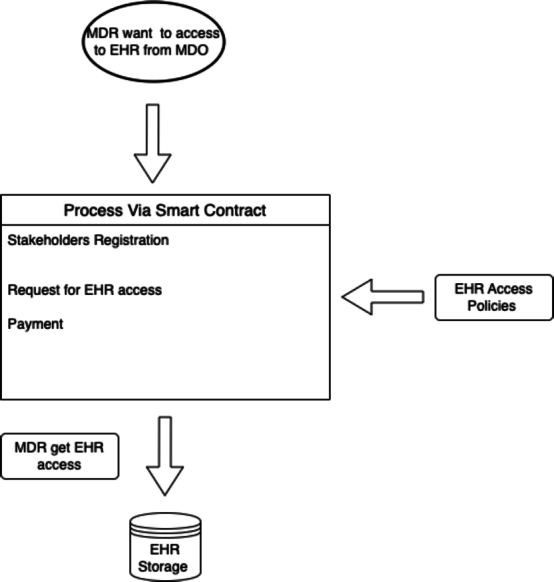
EHR Access Process.

#### 3.1.5 Access control policy verification for compliance auditing

**Figure 6:**
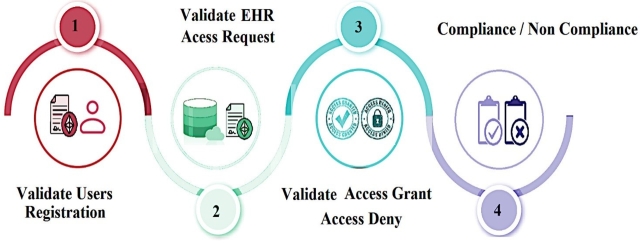
Procedure of Compliance Auditing.

**Figure 7:**
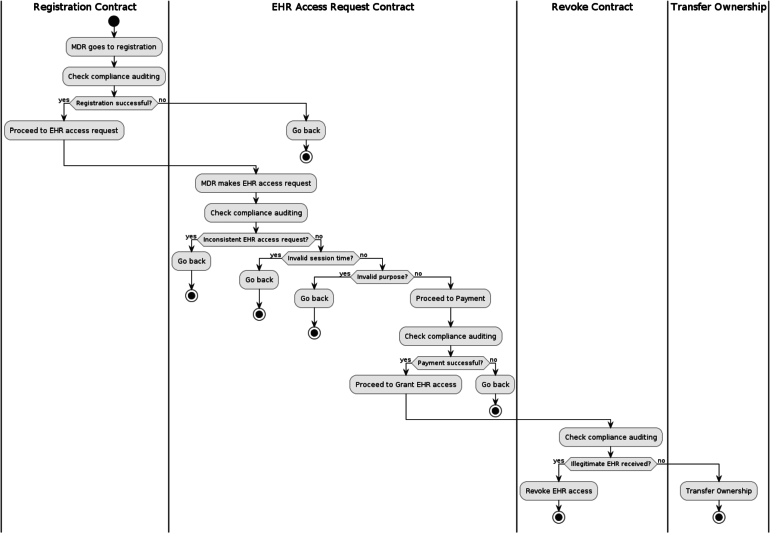
Flow of Compliance Auditing.

Compliance auditing, is a systematic process employed within a blockchain based *EHR* system. Compliance auditing entails a thorough examination and validation of *PBAC* policies, which define the rules governing the access to *EHR* data and the circumstances under which such access is permitted. This process involves several key stages, including verification during user registrations, access requests, access approvals or denials, and updates as shown in Fig. 6 and in Fig. 7. The system meticulously assesses these stages to ensure that they are in alignment with the established policies, confirming that the policies are being accurately and effectively implemented.

**Alignment with Standards:** The compliance auditing process ensures that the system's access control policies are in alignment with industry standards and regulatory frameworks. This alignment is important to demonstrate adherence to external rules and regulations.

**Error Handling:** Non-compliance with access control policies may lead to errors or access denials. These errors are integral to the auditing process, as they help to identify instances of non-compliance that need rectification.

This verification ensures that access granted to data requesters aligns with the stated research purpose and complies with the conditions specified in the access control policy as shown in Fig. 6 and in Fig. 7.

#### 3.1.6 Audit logging

Comprehensive audit logs are generated from two primary sources - the audit trail and the Access Control Policy Verification for Compliance Auditing. These logs enhance the robustness of our auditing system, facilitating thorough monitoring and investigation of access activities.

### 3.2 Design of smart contract

Smart contracts, supported by well-defined *PBAC* policies, provide both secure and transparent access to *EHR* between *MDO* and *MDR* in an efficient and trustworthy manner. It leverages advanced data structures, efficient mappings, purpose-driven enumerations, events and set of functions to deliver a reliable and auditable system. The functions within these contracts perform essential tasks. They manage user registration and verification to control access effectively. The functions also handle access requests, confirming eligibility, granting access, and recording auditing events. The functions facilitate ownership changes and ensure transparency through the emission of events that document ownership transitions. Enumerations, or predefined sets of named values, to define the various types of *EHR* and the purposes for which access is permitted. These enumerations enable the contract to accurately and efficiently categorize the data and regulate access to it. The contract employs mappings, which are data structures that map one set of values to another, to store critical information about the access control, access time, location, session time, and request timestamp of the *EHR* data. These mappings serve as essential data repositories, enabling the contract to effectively monitor and manage access to the *EHR* while preserving essential audit trail information like *UP*, *TC*, and *EM*. Lastly, the contract defines several events that are emitted whenever specific actions occur, such as when an access request is made, approved, or denied. These events enable the contract to trigger other actions, track activity, and provide an immutable record of all the relevant transactions.

#### 3.2.1 Registration contract

The Registration Smart Contract incorporates data structures, mappings, and purpose-driven enumerations to efficiently manage user registration, data storage, and access verification. It ensures compliance auditing through access control policy verification, supported by an audit trail mechanism encompassing vital components such as *UP*, *TC* details, and *EM*. Within this framework, the Registration Smart Contract defines a registration system where users can enroll through the *-signUp-* function, registering new users, and utilizing the *-isRegistered-* function to verify existing user registrations. This approach to user registration includes a detailed audit trail, encompassing user profiles among other essential information. The contract's functionality extends to the validation of requester through the *-validateRequester-* function, ensuring compliance with access control policies. The “enum Purpose” adds clarity to the purpose of each *EHR* request, contributing to compliance auditing through access control policy verification. The *-Requester-* struct captures details of requester, facilitating an audit trail that documents their actions and data access attempts comprehensively. Simultaneously, the *-Data-* struct, equipped with fields for payment and *-dataHash-*, ensures transaction recording and audit facilitation, incorporating key Audit Trail information, including *UP*, *TC* details, and *EM*.

The *-DataOwner-* struct serves as a repository for data owners, recording their activities and data sharing events, adding another layer to the audit trail. Efficient mappings play a role in the contract's auditing capabilities. The *-requesters-* mapping establishes a clear link between Ethereum addresses and *-requesters-* structs, facilitating an audit of requester registrations and access attempts. Similarly, the *-dataOwners-* mapping connects Ethereum addresses to *-dataOwners-* structs, providing a coherent audit trail for data owner actions and data sharing activities. This structured approach to mappings encompasses crucial Audit Trail information, including *UP*, *TC* details, and *EM*, ensuring a thorough compliance audit.

#### 3.2.2 Access request contract

The Access Request contract includes a *-requestAccess-* function that allows users to make a request for access to the *EHR*. This function verifies that the user has the necessary permissions and that the values submitted (such as the purpose, time period, location, and session) which are *TC* and are valid contributing to compliance auditing. It also ensures that the contract has sufficient balance to proceed with the transaction and that the user is registered on the registration contract as well as set the price between *MDR* and *MDO*. If all these conditions are met, the contract emits the AccessRequestMade event, ensuring compliance auditing, and then checks whether the user has permission to access the *EHR* for the specified purpose. If the user does not have permission, the contract emits the *-AccessDenied-* event. However, if the user does have permission, the contract emits the *-AccessGranted-* event, effectively granting the user access to the requested *EHR* while maintaining an audit trail and compliance auditing. Ownership changes from *MDO* to *MDR* management are facilitated through the *-setOwner-* function, which enables the contract owner to assign additional owners. These ownership changes are recorded through emitted events, establishing an audit trail that documents modifications and ownership transitions, ensuring compliance auditing via access control policy verification.

#### 3.2.3 Revoke contract

The Revoke Contract serves as a valuable tool for the better management of *EHR* data access on the blockchain, ensuring that only authorized users have the privilege to access and utilize legitimate *EHR*. Its primary purpose is to govern access to *EHR* data stored on the blockchain, a responsibility that extends to the configuration of its constructor function, which establishes the contract owner and incorporates two additional contracts as parameters: *-RegistrationContract-* and *-AccessRequest-*. This contract introduces two central functions: *-reportData-* and *-revokeData-*. The *-reportData-* function is accessible to users who wish to report instances of unauthorized or illegitimate *EHR* access. Conversely, the *-revokeData-* function is reserved for the contract owner's use, empowering them to revoke data access for *MDRs* who have paid for access to illegitimate *EHR*. To facilitate seamless financial transactions, the contract includes a *-payToContract-* function, contributing to compliance auditing through access policy verification. To bolster access control and maintain a comprehensive log of significant events, utilizing modifiers and events Notably, the *-onlyOwner-* modifier restricts access to specific functions, ensuring that only the *MDO* or authorized users possess the authority to invoke these functions. Additionally, the contract emits two critical events: *-DataRevoked-* and *-RequestDenied-*. These events serve as integral components of the compliance auditing process by verifying access policies and offering transparency into instances when a user's data access is revoked or when their access request is denied.

The Algorithm 1 provides a structured and systematic approach to logging and monitoring activities in a blockchain-based system, facilitating transparency, accountability, and auditing of critical actions and events. The algorithm begins by initializing an empty list “auditTrail” to store these entries.

**Algorithm 1:**
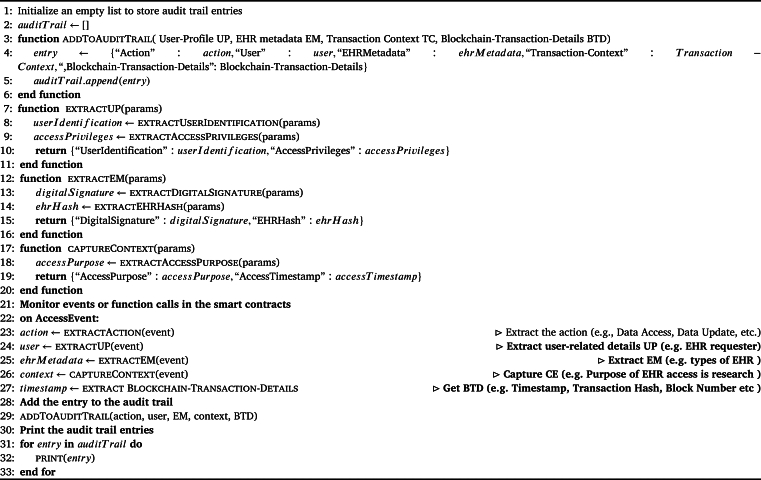
Audit Trail Algorithm.

The algorithm then uses function *-addToAuditTrail-*, and appends a new entry to the audit trail list. This entry contains various pieces of information, including the action performed, user details, EM, context, timestamp, transaction hash, and block number. This comprehensive entry ensures that each event or transaction's key aspects are recorded for auditing purposes. The algorithm defines functions to extract *UP*, *EM*, and capture contextual information *TC* from the input parameters, enabling the recording of pertinent details. The algorithm monitors events or function calls in smart contracts. Upon the occurrence of an *-AccessEvent-* it extracts essential information such as the action, user details, *EM*, context, timestamp, transaction hash, and block number. It then adds this information to the audit trail using the *-addToAuditTrail-* function.

The Algorithm 2 outlines a systematic approach to auditing compliance with access control policies in a blockchain-based system, focusing on Academic Research purposes. The algorithm begins by defining the access control policy, specifying conditions for different contracts (e.g., registration contract, accessRequest contract, revoke contract) and purposes (e.g., Valid Registration, Valid *EHR* access request and payment done, Violation Found). It then defines a function, *-verifyAccessControlPolicy-*, which evaluates log entries against the policy rules, determining whether access is compliant or not. Subsequently, the *-auditAccessControlCompliance-* function categorizes log entries as compliant or non-compliant based on policy verification. Given a set of log entries, the algorithm performs access control policy verification, segregates logs into compliant and non-compliant categories, and prints the results, providing a clear and structured approach to auditing and verifying access control policy compliance in the context of Academic Research purposes within a blockchain system.

**Algorithm 2:**
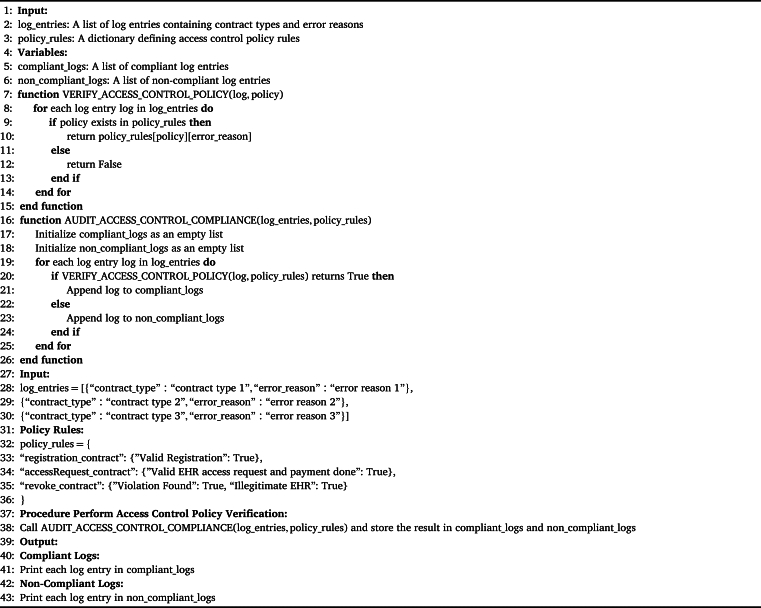
Auditing Compliance via Access Control Policy Verification.

The Algorithm 3, “Audit Logs Representation,” defines the structure and organization of audit logs within a system. It initializes a set, *AL*, to hold audit logs and then proceeds to populate it with individual log entries (log1, log2, ... logn). Each log entry adheres to a structured format, encompassing essential information such as the involved entity or user (useri), the accessed or modified resource (resourcei), the purpose of the access or operation (purposei, e.g., Academic Research), the timestamp of the log entry's creation (timestampi), the outcome of the access or operation (outcomei, e.g., Success, Failure), and a binary indicator of compliance with access control policies (Complianti, True/False). This representation ensures that audit logs are organized, standardized, and include critical details for monitoring and analysis of system activities.

**Algorithm 3:**
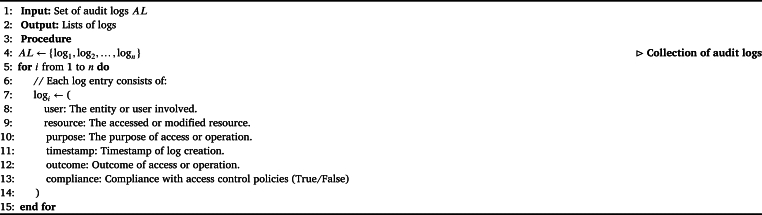
Audit Logs Representation.

## 4 Results and discussion

### 4.1 Audit trail results

The transaction details recorded on the blockchain, such as the transaction hash, sender address, and other parameters, form the audit trail. These details provide a traceable history and ensure accountability for the transaction. The use of blockchain technology ensures that the audit trail is tamper-proof and transparent, making it difficult to alter or manipulate the recorded information. Any attempts to modify or access the data without authorization can be easily detected.

**Figure 8:**
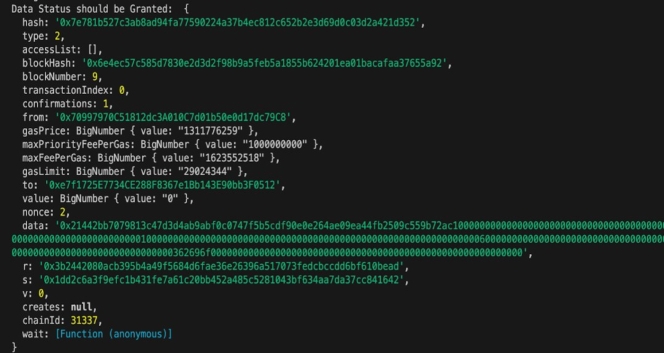
Details of Transactions EHR Access Grant.

Data Status and Grants: The audit trail depicted in Fig. 8 underscores the successful granting of *EHR* access. The *MDO* with the Ethereum address ‘0x70...9C8’ confirms the approval of access to the *MDR* identified by the address ‘0xe7f....F0512’ through a transaction hash (‘0x7e....352’), constituting a significant aspect of the *UP* within the audit trail. *EHR* metadata is represented by the ‘data’ field as ‘0x2144....00000’. Other details in transactions include gas consumption in the transaction, Nonce providing sequencing information, and digital signature components *(r,s,v)* verifying transaction authenticity. Chain *ID* offers insight into the designated blockchain network, the Block details indicate the transaction's position in the blockchain. The Value field, indicated as “0”, confirms the absence of financial transfers within the transaction.

### 4.2 Execution time

**Figure 9:**
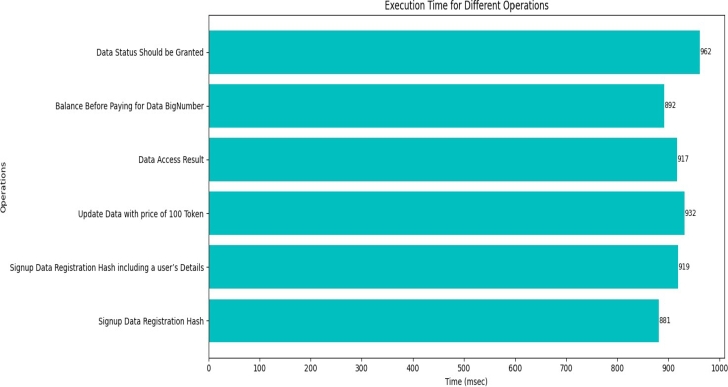
Execution Time of Transactions.

The term execution time in the context of smart contract transactions, it refers to the duration required to undergo processing and execution of transactions within the blockchain network. The Fig. 9 provides a visual representation of the varying execution times associated with different transactions. When tracing the back the auditing operation, the functions in smart contract “data status should be granted” and “update data with price of 100 token” takes more times to execute.

**Figure 10:**
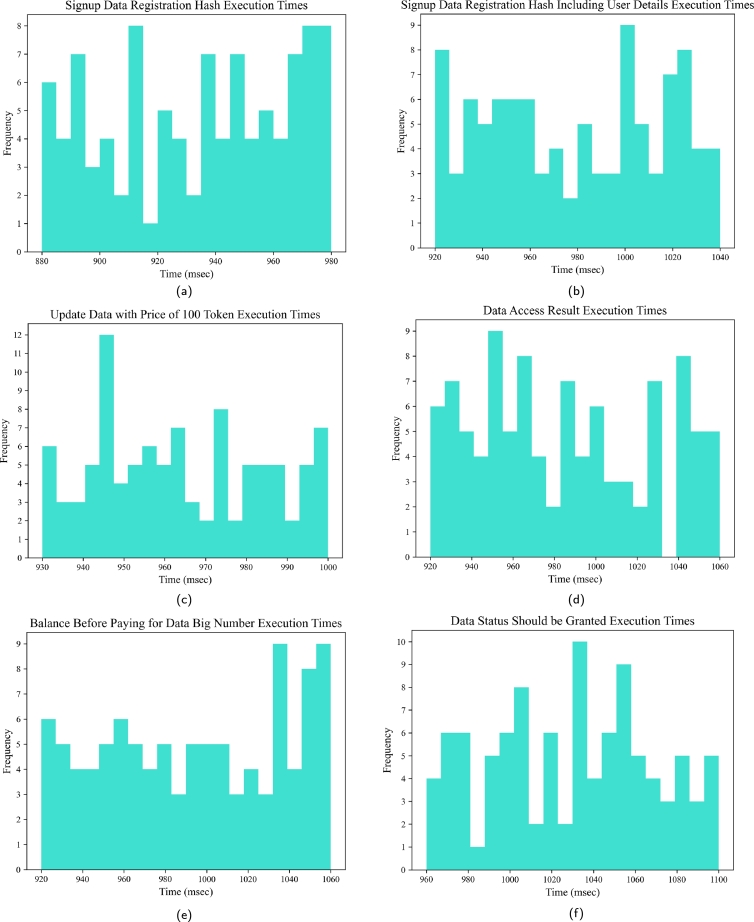
The Execution Time of 100 Iterations for (a): Signup Registration (b): Signup with user's details of 100 Iterations (c): Update Data with Price of 100 Token (d): Data Access Result (e): Balance Before Paying for Data (f): Data Status Should be Granted.

For a detailed and large scale visualization, we have conducted a simulation to assess the performance of various tasks within a healthcare system scenario involving 100 iterations. Each iteration represents the execution of these tasks and introduces a level of unpredictability, mirroring the dynamic nature of real-world user behavior. To capture this variability, we have generated execution times it randomly within specified ranges. The resulting histograms offer valuable visualizations, depicting the distribution of execution times across the 100 iterations This provides valuable insights into the system's performance under a wide range of user scenarios, aiding in the understanding of EHR access In the histogram of Fig. 10, the x-axis represents different time intervals (execution time ranges), and the y-axis shows the number of iterations falling within each time interval. The histogram in Fig. 10 (a) shows the range of execution time from 881 msec to 992 msec for each of the 100 iterations. The lowest execution time interval, from 881 msec to 884 msec, has a frequency of 6 iterations, indicating that 6 iterations had very quick execution times in this range during the registration process. On the other hand, the highest execution time interval in Fig. 10 (b), having 8 iterations, represents the longest execution times during registration. Similarly, “the Signup Data Registration Hash Include user's detail” in Fig. 10 (b) has lowest execution time interval, from 919 msec to 926 msec, while 8 iterations having the highest execution time interval from 943 msec to 952 msec and so on for the Fig. 10 (c), Fig. 10 (d), Fig. 10 (e), Fig. 10 (f).

#### 4.2.1 Gas cost

In Ethereum [39], gas cost represents the computational resources required to execute transactions and smart contracts on the blockchain. It determines the transaction fee users must pay for processing their actions, incentivizes miners, and acts as a security measure to prevent network abuse.

**Figure 11:**
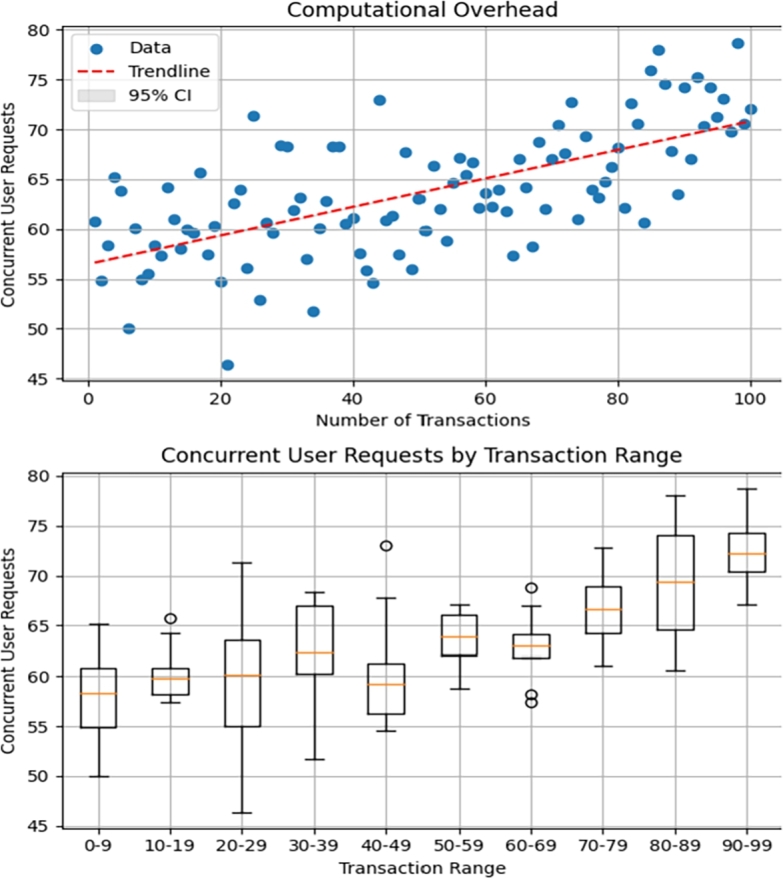
Computational Overhead.

The Table 2 presents the gas cost of a collection of transactions during audit trails operations executed within different contracts on the Ethereum blockchain, accompanied by their respective gas costs in Ether (*Eth* and their approximate equivalents in *US* dollars. These transactions encompass a range of actions, from data registration and updates to access permissions and balance inquiries. Notably, the gas costs associated with these transactions vary, indicating differences in computational complexity. For instance, during auditing registering data with user details results in a slightly higher gas cost compared to registering data with just its hash, reflecting the additional computational resources needed for processing the expanded data size. Similarly, transactions involving large numerical values, such as checking the balance before making a payment, tends to incur higher gas costs due to the computation or data handling involved. These gas costs are integral considerations in the realm of blockchain operations, impacting transaction fees and the overall efficiency of blockchain-based applications. See Fig. 11.

**Table 2:** Gas Cost of the transactions used in Different Contracts.

Transactions of Different Contracts	Gas Cost in US$
Signup Data Registration Hash	2.79
Signup Data Registration Hash including a user's Details	3.23
Update Data with Price of 100 token	5.4
Data Access Results	3.06
Balance Before Paying for Data Big Number Value	6.19
EHR Access Granted	3.24
Data Status Should be Granted	2.2

The figure demonstrates that computational overhead increases with auditing transactions as expected. The base computational overhead is set at 50, where *overhead_increase_rate* is set to 2. In the equation

(1) $$\text{concurrent}\_\text{user}\_\text{requests}=\text{base}\_\text{overhead}+\text{overhead}\_\text{increase}\_\text{rate}\times \sqrt{\text{transactions}}$$

the term $\text{overhead}\_\text{increase}\_\text{rate}\times \sqrt{\text{transactions}}$ represents the proportional increase in computational overhead as transactions increase. This indicates that as the number of transactions increases, the computational overhead escalates, exemplified by the scatter plot's trendline and the box plot's median values. For instance, with an intercept of around 53 and a slope of approximately 0.5, indicating the increase of computational overhead due to the increase of auditing transactions

### 4.3 Compliance auditing via access control policy verification

**Figure 12:**
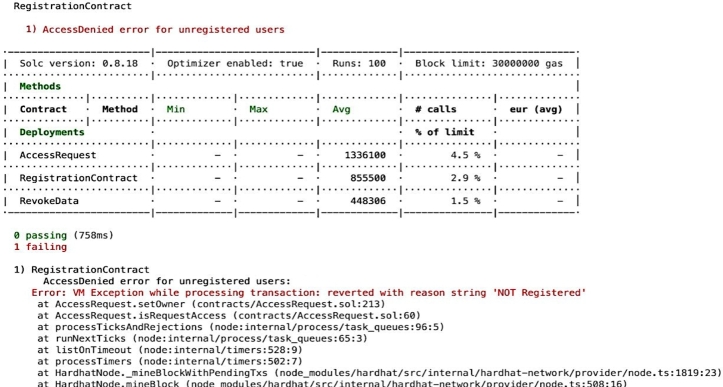
Unable to Proceed Next Due to Unsuccessful Registration and Validation.

**Figure 13:**
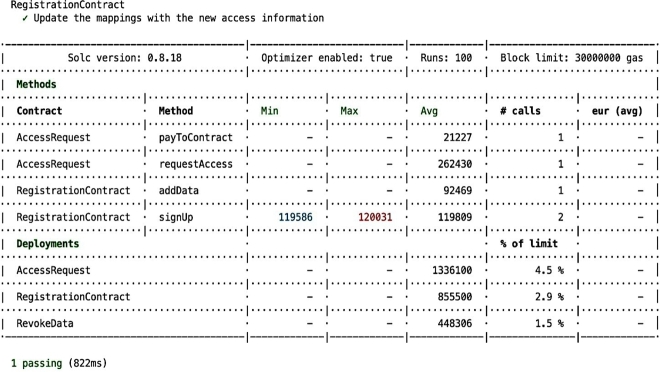
Update the Data When New Access Information is Added.

**Figure 14:**
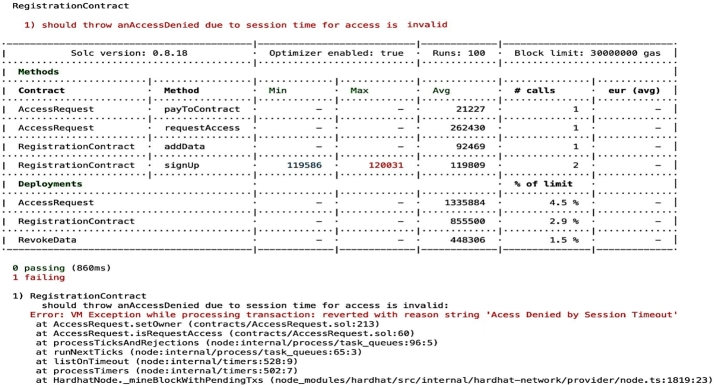
Access Denied when an Entity Tries to get Access At Invalid Session Time.

**Figure 15:**
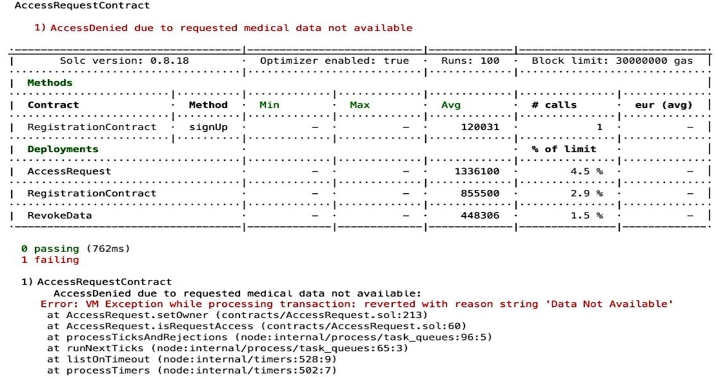
Access Denied Due to Requested Inconsistent EHR.

**Figure 16:**
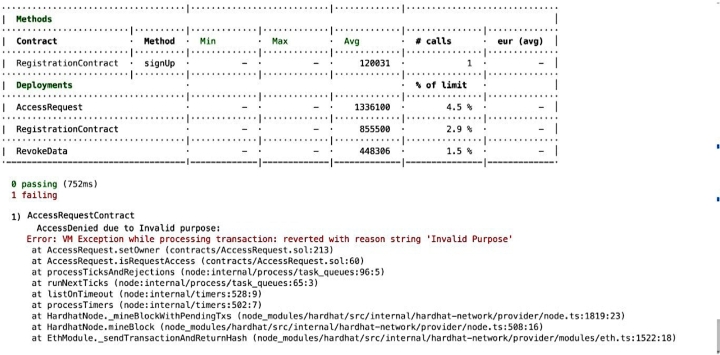
Access Denied Due to Invalid Purpose.

**Figure 17:**
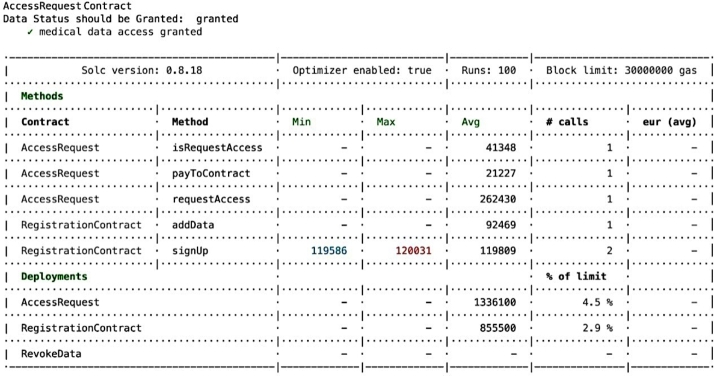
EHR Access Granted after Payment.

**Figure 18:**
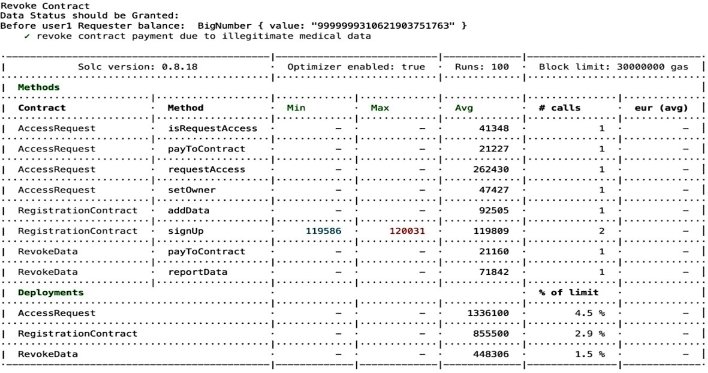
Revoke Payment When EHR received is Illegitimate.

Compliance auditing involves carefully checking if the system adheres to validation checks and complies with the access control policies It is essential to verify and check whether applied policies are functioning effectively, ensuring adherence to the policies and overall compliance. For example, it is important to validate that unauthorized entities cannot gain access, unregistered users are prevented from entering the system, and authorized individuals are only granted access to permissioned *EHR* resources, as depicted in the Fig. 12, Fig. 13, Fig. 14, Fig. 15, Fig. 16, Fig. 17 and in Fig. 18 to ensure compliance.

The smart contract for compliance auditing is written in Solidity version 0.8.18 and is optimized for performance, having undergone 100 rounds of testing. The smart contract, designed to consume a maximum of 3,000,000 units of gas, which is the transaction fuel in the blockchain world, underwent 100 rounds of testing for optimization and performance. During deployment, the RegistrationContract consumed 855,500 gas, equivalent to 2.9 During the registration phase, verifying access control policies involves confirming the process for allowing authorized individuals, which includes confirming their name, unique *ID*, and blockchain address thus contributing to compliance auditing by validating and verifying *PBAC* policies. Ensuring that the individual requesting access aligns with the one who initiated the request is crucial. This confirmation of *EHR* ownership involves cross-referencing the initiator of the request using a specialized function called message.sender(). For the case where an entity's verification falls short typically due to an incomplete registration or incorrect details, the system generates an error message, as seen in Fig. 12. The denial of access ensures that access control policies are working correctly aligning with the compliance auditing.

For the case where users require updating their information, the compliance auditing system facilitates and accommodates such actions. The Fig. 13 illustrates when a user initiates updates to their registration or adjusts their access permissions in response to another *EHR* access request. In this process, when a user makes changes to their registration, such as modifying access preferences or responding to a new access request, the system updates the mapping associating user profiles with *EHR* metadata and access permissions. These updates ensure that the user's access information aligns with their preferences and any new access requirements continuing the process of compliance auditing by validating and verifying *PBAC* policies.

For the cases where unauthorized entities attempt to gain access or authorized entities seek access after a session timeout, the system enforces measures to prevent such actions in order to uphold compliance standards. The Fig. 14 visually represents where access is denied to an entity attempting to gain entry during an unauthorized session time whether due to session timeout or an attempt at the wrong moment. This depiction highlights the robustness of our access control mechanism, which not only enforces session-based access restrictions but also aligns seamlessly with compliance auditing standards. Our access control mechanism meticulously verifies access policies, ensuring that only legitimate access attempts made within valid timeframes and in strict adherence to established policies are granted. This dual-layered approach fortifies the security of our *EHR* system while upholding the standards of regulatory compliance. It exemplifies our commitment to safeguarding sensitive health data and maintaining the integrity of our system. For the case where an entity attempts to access *EHR* assets that were neither authorized nor requested, an error message will be generated indicating that the requested *EHR* is not available shown in Fig. 15 contributing to the compliance auditing by ensuring that access attempts align with established permissions and requisitions.

Compliance auditing plays an important role in ensuring that the proposed framework functions effectively in denying access to requested resources that are not explicitly stated in their intended purpose. The similar case as previous, when an individual tries to gain access to a resource for a purpose that wasn't approved for instance an entity who had initially registered with the explicit purpose of “accessing *EHR* for research” attempts to access for “clinical treatment” purposes then an error message of “invalid purpose” will be displayed and access will be denied as depicted in Fig. 16 aligning with compliance auditing standards. This mechanism is in place to regulate access by preventing unauthorized entities from accessing resources not designated for their intended purpose.

For the cases when *MDR* necessitates making trade-offs with *MDO*, and subsequent payment verification is successfully completed, the compliance framework is adaptable to accommodate such transactions as shown in Fig. 17. The accessRequest contract emits the AccessGranted event, grant the access to the *MDR* to the specific *EHR* while ensuring access control policy verification and compliance auditing are in place.

Compliance auditing closely monitors and verifies the accuracy of payment transactions involving, ensuring that the financial aspect aligns with the intended purpose and valid recipients. For the cases, where a *MDR* request necessitates payment revocation subsequent to the receipt of illegitimate *EHR*, compliance measures are flexible enough to accommodate such scenarios, as illustrated in Fig. 18 The *-payToContract-* function in the Revoke Contract facilitates payment revocation. The figure highlights that payment has been successfully revoked from the *MDR* who received illegitimate *EHR*, the compliance auditing process helps make sure the financial part of this framework is reliable and strong.

#### 4.3.1 Execution time

**Figure 19:**
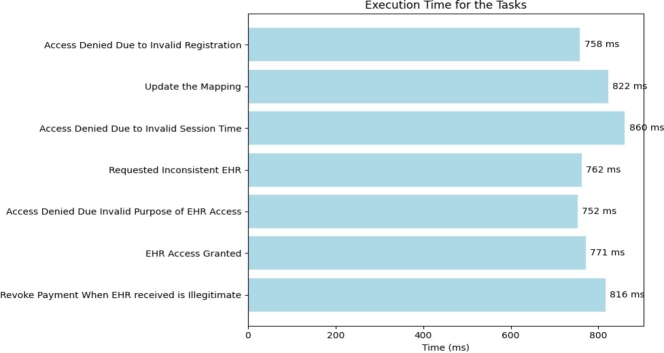
Execution Time.

The smart contract execution time of the various above mentioned tasks during the verification of access control policy are shown in Fig. 19.

**Figure 20:**
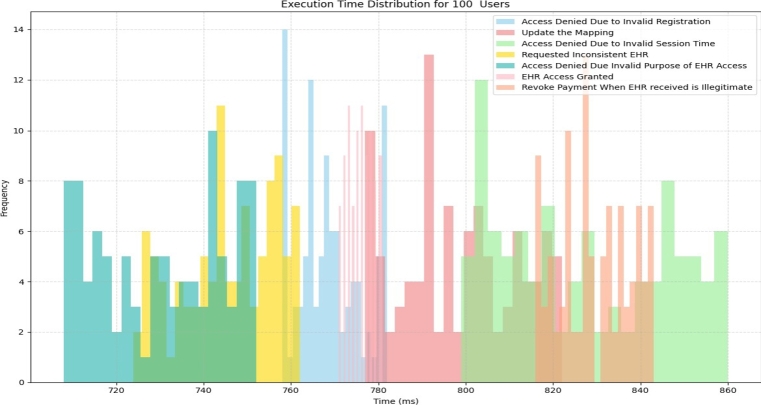
Execution Time for 100 Iterations.

For the detailed and large scale visualization, we have simulated the execution times of various tasks in a scenario involving 100 iterations as presented in Fig. 20. These tasks include actions such as “Access Denied Due to Invalid Registration,” “Update the Mapping,” “Access Denied Due to Invalid Session Time,” “Requested Inconsistent *EHR*,” “Access Denied Due to Invalid Purpose of *EHR* Access,” “*EHR* Access Granted,” and “Revoke Payment When *EHR* Received is Illegitimate.”

Each iteration of these tasks results in varying execution times, mirroring the inherent unpredictability of real-world user behavior. These execution times have been randomly generated within specified ranges to replicate this variability. The resulting histogram offers informative visualizations of the distribution of execution times across the 100 iterations, providing insights into the system's performance under a range of user scenarios. For instance, in the figure, we observe the occurrence of the “Access Denied Due to Invalid Purpose” event with varying frequencies and distinct execution times. Specifically, when we see an execution time of 652 milliseconds accompanied by 8 iterations, it signifies that the action of users attempting to access the system with purposes not aligned with the predefined criteria has occurred on 8 different occasions.

The histogram highlights that “Access Denied Due to Invalid Session Time” is the predominant event, indicating that a significant number of entities attempted access with incorrect session times. Conversely, “Access Denied Due to Invalid Registration” exhibits the lowest frequency among the events recorded.

#### 4.3.2 Gas cost

The gas cost associated with transactions of smart contracts during compliance auditing is shown in Table 3. In the Registration Contract, operations such as “Access Denied Due to Invalid Registration” and “Update the Mapping” consume 0.002651 *ETH* (approximately 4.23 *USD*) and 0.002231 *ETH* (approximately 3.56 *USD*) in gas costs, respectively shown in Table 3. Similarly, in the AccessRequest Contract, “*EHR* Access Granted” proves to be a relatively efficient operation with a gas cost of 0.0001567 *ETH* (approximately 0.25 *USD*). Conversely, “Revoke Payment” within the Revoke Contract requires 0.002632 *ETH* (approximately 4.20 *USD*) in gas costs. These gas cost insights are crucial for users and developers to optimize Ethereum transactions and smart contract interactions while considering both computational resource consumption and associated financial implications.

**Table 3:** Gas Cost of the Function/Mapping used in Contracts.

Contract	Function/Mapping	Gas Cost in Eth	Gas Cost in US$
Registration Contract	Access Denied Due to Invalid Registration	0.002651	4.23
Registration Contract	Update the Mapping	0.002231	3.56
Registration Contract	Access Denied due to Invalid Session Time	0.002757	4.4
Registration Contract	Requested Inconsistent EHR	0.003798	6.06
AccessRequest Contract	Access Denied Due to Invalid Purpose of EHR Access	0.00262	4.19
AccessRequest Contract	EHR Access Granted	0.0001567	6.24
Revoke Contract	Revoke Payment	0.002632	4.2

#### 4.3.3 Communication complexity overhead

In evaluating the communication complexity of our EHR access system, we use the following expression:

The total communication complexity $C_{c}$ is given by:

$$C_{c}=N\cdot S_{t}$$

where:

• *N* represents the number of transactions involved in the EHR access process.
• $S_{t}$ denotes the size of each transaction, which includes both the metadata and the EHR segment.

To analyze the proportionality constant $k_{c}$ for communication overhead:

$$C_{c}=k_{c}\cdot N$$

• **Proportionality Constant (**$k_{c}$**)**: This constant accounts for the fixed communication overhead per transaction, including the metadata size and the average size of EHR segments.

For our system, the metadata and EHR segment sizes were analyzed based on standard sizes and sample records, ensuring accurate representation of communication overhead.

#### 4.3.4 Computational complexity overhead

The total computational complexity $C_{p}$ is given by:

$$C_{p}=H\cdot h+E\cdot e$$

where:

• *H* is the number of hashing operations required for security measures.
• *h* denotes the time complexity of a single hashing operation.
• *E* represents the number of smart contract executions.
• *e* denotes the time complexity of a single smart contract execution.

To analyze the proportionality constant $k_{p}$ for computational overhead:

$$C_{p}=k_{p}\cdot (H+E)$$

• **Proportionality Constant (**$k_{p}$**)**: This constant includes the fixed computational overhead per operation, encompassing both hashing operations and smart contract executions.

For our system, the hashing operations and smart contract executions were analyzed based on standard benchmarks and gas cost analysis, ensuring accurate representation of computational overhead.

By analyzing the small constants of proportionality in both communication and computational complexity, we provide a detailed understanding of the overheads involved in our system. This analysis demonstrates the theoretical improvement in terms of efficiency and provides a clearer picture of the system's performance.

#### 4.3.5 Comparative analysis of response time for access control

**Figure 21:**
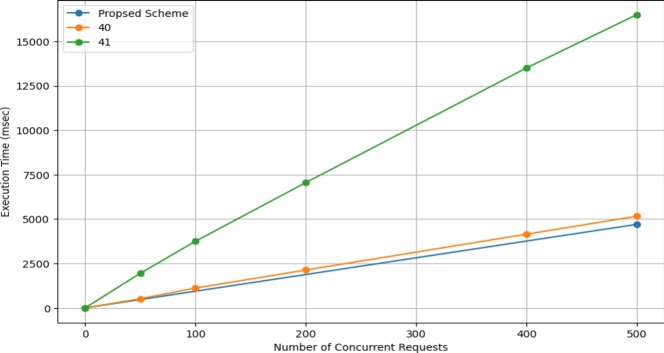
Comparison with Studies.

The time taken from system initialization to processing an access request is illustrated in Fig. 21. We compare our approach with the latest in the field, referenced as [40] and [41]. The graph distinctly shows that our system's response time is notably superior to the others presented.

### 4.4 Audit log

The audit logs are created by extracting and compiling data from the audit trail and access control policy verification processes.

**Figure 22:**
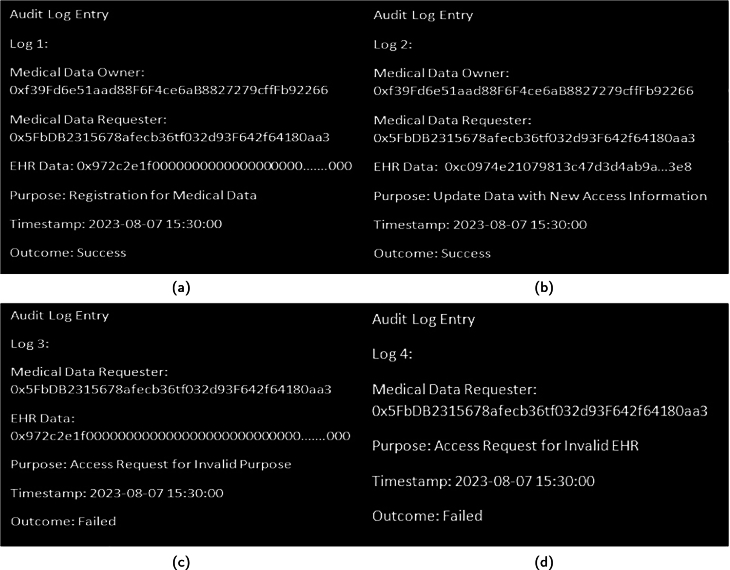
Audit Logs for (a): Signup Registration (b): Update Data (c): Access Failure Due to Invalid Purpose (d): Access Failure Due to Invalid EHR.

These logs represent a condensed and user-friendly representation of the detailed activities and decisions recorded within the smart contracts, offering a high-level overview of access requests, outcomes, and related information. Together, these components form an approach to managing and monitoring data access in the described system for audit logs to ensure data security and accountability. In the provided audit log entries in Fig. 22 (a), Fig. 22 (b), Fig. 22 (c), Fig. 22 (d) a subset of data access events has been described. In the first log entry, a successful registration for medical data took place, involving the medical data owner and requester. The purpose was to “Update Data with New Access Information,” resulting in a successful outcome. The next entry indicates a failed access request due to an “Invalid Purpose.” Here, the requester attempted to access medical data with an inappropriate purpose, resulting in a “Failed” outcome. Lastly, an access request for an “Invalid *EHR*” by the same requester resulted in another “Failed” outcome. These audit logs serve as a record of data access events, highlighting both successful and failed attempts, as well as the reasons behind the failures, such as invalid purposes or *EHR*

### 4.5 Access pattern and data analysis

In these studies, we aimed at improving compliance auditing within the context of blockchain based audit trails and access control policies for *EHR* access, we faced a significant hurdle, the scarcity of accessible access control entry datasets from real-world *EHR* systems. Understanding the importance of such data for our work, we took on the task of creating synthetic data that could closely resemble real-world scenarios.

We used a dataset source [42] as our reference and carefully generated synthetic data that mimicked genuine access control situations in *EHR*. Within this synthetic dataset, we created a heatmap, which is a graphical representation. This heatmap shows data access permissions for various roles within the *EHR* environment. These roles include doctors, radiologists, clinical staff, medical researchers, *IT* staff, and administrative personnel.

**Figure 23:**
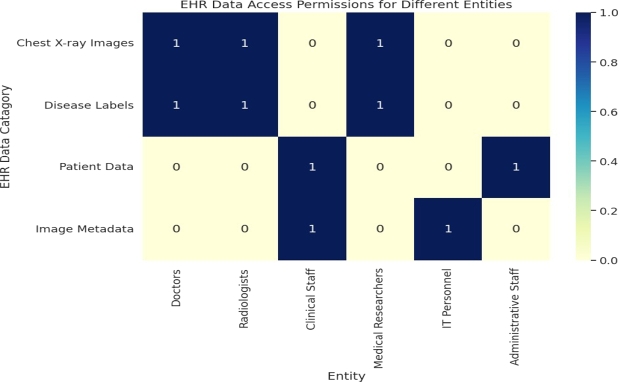
EHR Access Permissions for Different Entities.

We see by compliance verification regarding who can access the metadata of *EHR*, for example doctors are restricted from accessing this data as it is irrelevant to their role, while *IT* staff has the necessary permissions to do so as shown in the Fig. 23. In the heatmap in Fig. 23, simple ‘0’ and ‘1’ values to convey whether permission exists for a specific role to access a particular category of *EHR* data. A ‘0’ indicates that no permission is granted, while a ‘1’ means access is allowed in line with established rules. Doctor has only permission to access the *EHR* data which are labeled “Disease Labels” and “Chest X-ray Images” while the image metadata such as image format, image orientation and patient information such as billing information, consent form can't be accessed by doctors.

Access to image metadata is restricted to *IT* personnel for tasks like maintenance, uploads, downloads, and other essential functions. This limitation enhances data security, ensures privacy compliance, maintains data integrity, and facilitates accurate audit trails. Administrative staff has access to patient data, including billing information and consent forms, while following strict access control measures. This approach enhances data security, ensures privacy compliance, and allows for the maintenance of detailed audit trails to track any changes or accesses to sensitive patient information.

It is important that this heatmap doesn't reflect the actual outcomes of our smart contract-based auditing and access control processes. Instead, it serves as a visual tool to help explain the fundamental concepts of *EHR* data access permissions and the world of compliance auditing that is at the core of our research.

**Figure 24:**
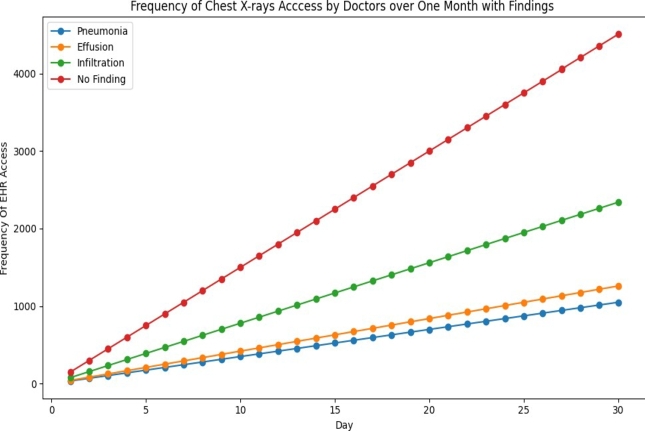
Frequency of Access by Doctors Over One Month with Different Findings.

From the dataset, we focus only the access permission, access details of only “doctors” in the study for auditing purpose. The Fig. 24 provides a compelling representation of the frequency of different findings, including “Pneumonia,” “Effusion,” “Infiltration,” and “No Finding,” within chest X-ray data over a span of 30 days. The prominence of “No Finding” underscores the frequency of routine, non-diagnostic X-ray procedures, where no abnormalities are anticipated. In contrast, “Infiltration,” “Effusion,” and “Pneumonia” signify conditions demanding closer clinical attention and, consequently, specialized access. This distribution, which aligns with typical patterns in medical imaging datasets, underscores the importance of tailoring auditing mechanisms to this data landscape. It's a call to action, emphasizing comprehensive access control policies and audit trail mechanisms, especially concerning access to findings such as “Infiltration,” “Effusion,” and “Pneumonia.” It emphasizes the need to allocate resources towards identifying and monitoring access patterns associated with these less frequent yet clinically relevant findings.

**Figure 25:**
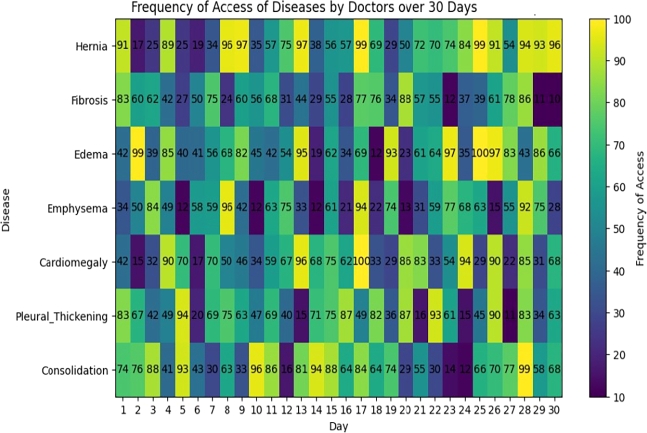
Frequency of Access by Doctors Over One Month with Different diseases.

**Figure 26:**
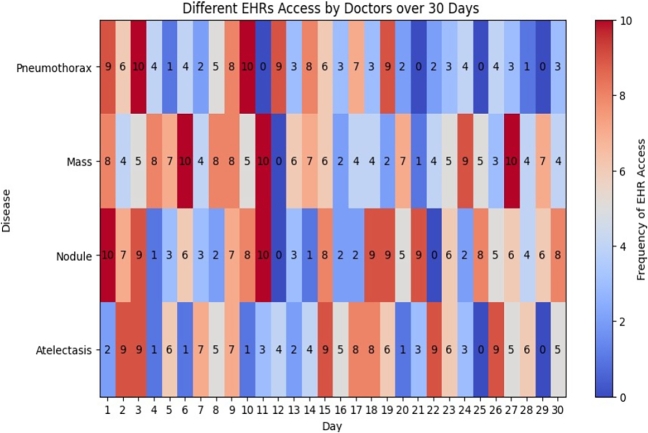
Frequency of Access by Doctors Over One Month with Different Findings.

The heatmaps presented in Fig. 25 and Fig. 26 provide a visual depiction of how frequently doctors accessed various diseases over a span of 30 days. Each cell within these heatmaps symbolizes the quantity of accesses to a particular disease on a given day. Notably, high values within the heatmap cells, as exemplified by ‘Hernia’ and ‘Emphysema,’ signify heightened access rates, possibly indicating their clinical significance. Conversely, low values, including zeros, indicate infrequent access, reflecting stringent privacy and security measures safeguarding patient data.

The heatmap captures the dynamic nature of data access, underscoring variations in access frequencies across diseases. It conveys the intricacies of the data access landscape and underscores areas necessitating auditing and policy enforcement. Specifically, the presence of low or zero access values for certain diseases offers operational advantages, permitting more efficient resource allocation for auditing. Resources and attention can be strategically directed toward scrutinizing access to diseases with higher frequencies or specific concerns.

The heatmap serves as an illustrative example of data access monitoring and can be a starting point for discussions on more sophisticated audit trail mechanisms and access control policies.

**Theorem: Security Proof for EHR Access Control Policies**

Consider an *EHR* system with *PBAC* policies enforced through a blockchain-based audit trail. Let S denote the system's state space, A the set of actions, and O the set of possible outcomes. Define the access control policy as a function PBAC:S×A→{0,1}, where PBAC(s,a)=1 indicates that action *a* is permitted in state *s*, and PBAC(s,a)=0 otherwise

1. **Confidentiality:**
  • The access control policy *PBAC* ensures confidentiality by ensuring that only authorized entities can access *EHR*. For any state *s* and action *a*, the probability of unauthorized access is minimized:
    $$P[\text{Unauthorized Access}]=\sum_{s\in S}\sum_{a\in A}P(s)\cdot P(a|s)\cdot (1-\text{PBAC}(s,a))$$
  • The given probability is reduced by the correct implementation of *PBAC*, the accuracy of user identification, and the precise allocation of access privileges.
2. **Integrity:**
  • The audit trail algorithm ensures the integrity of logged actions. Let AT:S×A→Audit Logs be the audit trail function. The probability of a tampering event is minimized:
    $$P[\text{Tampering}]=\sum_{s\in S}\sum_{a\in A}P(s)\cdot P(a|s)\cdot \text{AT}(s,a)$$
  • The correctness of AT and its resistance to adversarial manipulation ensure the integrity of the audit trail.
3. **Availability:**
  • The availability of the system is guaranteed by the resilience of the underlying blockchain network and the prevention of denial-of-service *(DoS)* attacks. Let BN:S→{0,1} be the blockchain network state function, and DoS:S→{0,1} be the *DoS* attack detection function. The system's availability is expressed as:
    $$P[\text{System Availability}]=\sum_{s\in S}P(s)\cdot \text{BN}(s)\cdot \text{DoS}(s)$$

**Assumptions:**

1. **Honest Majority:**
  • The majority of entities in the system behave honestly, reducing the probability of malicious behavior:
    P[MaliciousBehavior] is minimal.
2. **Correct Implementation:**
  • The *PBAC* policies and the audit trail function AT are correctly implemented, ensuring their reliability:
    P[CorrectImplementation]=1.
3. **Blockchain Resilience:**
  • The blockchain network guarantees Byzantine Fault Tolerance, ensuring its resilience to adversarial attacks:
    P[BlockchainResilience]=1.

The security properties of the *EHR* access control policy are proven to uphold confidentiality, integrity, and availability, ensuring the system's robustness against unauthorized access, tampering, and disruptions.

### 4.6 Comparative analysis

The Table 4 offers a comparative analysis of auditing schemes of various studies in the context of blockchain technology. The table presents their focus on access control policies, auditing mechanisms, blockchain platforms, performance evaluation, application domains, and payment considerations. Notably, several studies, including [33], [34], [26], and [29], do not place a strong emphasis on access control policies. In contrast, reference [32] introduces auditing in the context of Hyperledger Fabric, while the proposed approach in the last row integrates *XACML* policies. While payment aspects are mostly overlooked in all the studies listed in table except the [21] and the proposed scheme.

**Table 4:** Comparative Analysis with Other Studies.

Reference	Access Control Policies	Audit	Blockchain Platform	Performance Evaluation	Field	Payment
[33]	No	Audit Trails Logs	No	Yes	EHR	No
[34]	No	Audit Logs	No	Yes	EHR	No
[26]	No	Audit Logs	H. Fabric	No	EHR	No
[29]	Yes / XACML	Auditing	H. Fabric	Yes	EHR	No
[21]	No	Auditing Data	Ethereum	Yes	IOT	Yes
[30]	No	Auditing Management	Quorum	Yes	IOT	No
[20]	No	Data Auditing	Ethereum	Yes	Network Storage	No
Proposed	YES / Purpose Based	Audit Trails, Compliance Auditing, Audit Logs	Ethereum	Yes	EHR	Yes

Our proposed approach appears to be comprehensive and robust, particularly when compared to the listed studies in the table. By incorporating three types of auditing (Audit Trails, Compliance Auditing, Audit Logs), *PBAC* policies, and payment functionalities our approach addresses critical aspects of blockchain implementation in *EHR*.

### 4.7 The experiment setup information

The experiments of this scheme were conducted on macos ventura version 13.2.1 with Apple M1 Pro i9 @3.20 GHz and 16GB RAM. We used solidity 0.8.18 latest version with hardhat also used hardhat optimizer enabled with 100 and deployed it on hardhat local network, ganache network and *BSC* testnetwork to verify all code on *BSC* explorer to show transparently all codes.

## 5 Conclusion and future work

In this study, we presented a novel approach to auditing the *EHR* access, leveraging blockchain and smart contracts technology with *PBAC* policies. We have presented three key three main areas of auditing in the context of *EHR* access: audit trails, verification of access policies for compliance auditing, and the generation of audit logs. The results of audit trails trace back the details of *EHR* access for examples, entities involved, purpose, the time of access etc. Compliance auditing ensures *PBAC* policies are functioning correctly while audit logs record all *EHR* access activities. Towards the end, we explored deriving data-driven insights through systematic analysis of *EHR* access auditing to understand data access permissions better.

### 5.1 Limitations

#### 5.1.1 Security models for adversaries

Although our study the blockchain-based auditing for EHR access, there is significant limitation. The inclusion of strong security models is imperative for effectively addressing the diverse array of security threats facing EHRs and audit trials, particularly those stemming from computational complexities. A secure storage model should rigorously establish computational intractability within the UC model framework, by defensing against potential adversaries.

#### 5.1.2 Storage free-riding attacks

Our study did not extensively focus on the analysis of malicious behavior exhibited by *MDR* entities. In the case when malicious *MDR* collaborate to expose patients sensitive information, there exists a vulnerability to storage free-riding attacks on stored audit trails. Addressing this risk demands the implementation of robust authentication and encryption mechanisms, complemented by the deployment of effective mitigation strategies.

#### 5.1.3 Blockchain network latency

Latency in blockchain networks may affect the real-time nature of *EHR* access auditing. Delays in transaction confirmation and block propagation could impact the timeliness of audit trail updates

### 5.2 Future work

#### 5.2.1 Developing a threat model

The current study primarily focuses on auditing of *EHR* access, however there is necessity of providing a strong threat model. Our future work is to create a strong threat model and explaining how our solution can resist attacks. This entails the development of a detailed threat model and subsequent testing to evaluate the resilience of our solution against various threat scenarios. Through these efforts, we aim to strengthen the security posture of healthcare systems and enhance their ability to mitigate cybersecurity risks effectively.

#### 5.2.2 51% attacks

While our current focus revolves around *EHR* auditing, it's essential to recognize the looming threat of 51% attacks in blockchain technology. As part of future exploration, developing computationally infeasible models to mitigate such threats are important. This could entail refining existing consensus mechanisms or delving deeper into cryptographic techniques tailored specifically to address this vulnerability.

#### 5.2.3 DAO attacks

Another future research is the investigation of vulnerabilities within smart contracts that could potentially lead to *DAO* attacks. Developing methods to strengthen the security of smart contracts is paramount in this regard, with considerations such as implementing multi-signature authentication and time-lock mechanisms to mitigate these threats.

## Funding

The research was conducted with the financial support of 10.13039/501100001602Science Foundation Ireland under Grant Agreement No. 13/RC/2106_P2 at the ADAPT SFI Research Centre at University College Dublin. ADAPT, Science Foundation Ireland funds the SFI Research Centre for AI-Driven Digital Content Technology through the SFI Research Centres Program, and it has also received support from the 10.13039/501100005089Beijing Natural Science Foundation (No. IS23054).

## CRediT authorship contribution statement

**Faheem Ullah:** Validation, Software, Resources, Project administration, Methodology, Investigation, Formal analysis, Data curation, Conceptualization. **Jingsha He:** Supervision. **Nafei Zhu:** Supervision. **Ahsan Wajahat:** Data curation, Conceptualization. **Ahsan Nazir:** Investigation, Formal analysis. **Sirajuddin Qureshi:** Resources, Project administration. **Muhammad Salman Pathan:** Validation, Methodology. **Soumyabrata Dev:** Funding acquisition.

## Declaration of Competing Interest

The authors declare that they have no known competing financial interests or personal relationships that could have appeared to influence the work reported in this paper.

## Data Availability

The source code used in this study is available in a public repository at GitHub. The code has been verified at BscScan. The dataset utilized, which includes access control patterns, is publicly accessible at GitHub.

## References

[1] Almaghrabi Nada Saddig, Bugis Bussma Ahmed (2022). Patient confidentiality of electronic health records: a recent review of the Saudi literature. Dr. Sulaiman Al Habib Med. J..

[2] Wu Guangjun, Wang Shupeng, Ning Zhaolong (2021). Blockchain-enabled privacy-preserving access control for data publishing and sharing in the Internet of medical things. IEEE Int. Things J..

[3] Mishra Rahul, Ramesh Dharavath, Edla Damodar Reddy, Qi Lianyong (2022). Ds-chain: a secure and auditable multi-cloud assisted ehr storage model on efficient deletable blockchain. J. Ind. Inf. Integr..

[4] Holmes John H., Beinlich James, Boland Mary R., Bowles Kathryn H., Chen Yong, Cook Tessa S., Demiris George, Draugelis Michael, Fluharty Laura, Gabriel Peter E. (2021). Why is the electronic health record so challenging for research and clinical care?. Methods Inf. Med..

[5] Aguirre Roboam R., Suarez Orlando, Fuentes Mailenys, Sanchez-Gonzalez Marcos A. (2019). Electronic health record implementation: a review of resources and tools. Cureus.

[6] Ettaloui Nehal, Arezki Sara, Gadi Taoufiq (2023). The International Conference on Artificial Intelligence and Smart Environment.

[7] U.S. Congress and Ways and Means Committee (2009).

[8] Bakare Seun Solomon, Adeniyi Adekunle Oyeyemi, Akpuokwe Chidiogo Uzoamaka, Eneh Nkechi Emmanuella (2024). Data privacy laws and compliance: a comparative review of the eu gdpr and usa regulations. Comput. Sci. IT Res. J..

[9] Bonyuet Derrick (2020). Overview and impact of blockchain on auditing. Int. J. Digit. Account. Res..

[10] Gadekallu Thippa Reddy, Wang Weizheng, Yenduri Gokul, Ranaweera Pasika, Pham Quoc-Viet, da Costa Daniel Benevides, Liyanage Madhusanka (2023). Blockchain for the metaverse: a review. Future Gener. Comput. Syst..

[11] Wu Guangjun, Wang Shupeng, Ning Zhaolong, Zhu Bingqing (2021). Privacy-preserved electronic medical record exchanging and sharing: a blockchain-based smart healthcare system. IEEE J. Biomed. Health Inform..

[12] Ganiga Raghavendra, Pai Radhika M., Sinha Rajesh Kumar (2020). Security framework for cloud based electronic health record (ehr) system. Int. J. Electr. Comput. Eng..

[13] Keshta Ismail, Odeh Ammar (2021). Security and privacy of electronic health records: concerns and challenges. Egypt. Inform. J..

[14] Basil Nduma N., Ambe Solomon, Ekhator Chukwuyem, Fonkem Ekokobe, Nduma Basil N., Ekhator Chukwuyem (2022). Health records database and inherent security concerns: a review of the literature. Cureus.

[15] Tariq Noshina, Qamar Ayesha, Asim Muhammad, Khan Farrukh Aslam (2020). Blockchain and smart healthcare security: a survey. Proc. Comput. Sci..

[16] Li Jiguo, Yan Hao, Zhang Yichen (2018). Certificateless public integrity checking of group shared data on cloud storage. IEEE Trans. Serv. Comput..

[17] Rao Lu, Zhang Hua, Tu Tengfei (2017). Dynamic outsourced auditing services for cloud storage based on batch-leaves-authenticated merkle hash tree. IEEE Trans. Serv. Comput..

[18] Azaria Asaph, Ekblaw Ariel, Vieira Thiago, Lippman Andrew (2016). 2016 2nd International Conference on Open and Big Data (OBD).

[19] Zheng Zibin, Xie Shaoan, Dai Hong-Ning, Chen Xiangping, Wang Huaimin (2018). Blockchain challenges and opportunities: a survey. Int. J. Web Grid Serv..

[20] Xu Ronghua, Chen Yu, Blasch Erik, Chen Genshe (2018). 2018 IEEE International Conference on Internet of Things (iThings) and IEEE Green Computing and Communications (GreenCom) and IEEE Cyber, Physical and Social Computing (CPSCom) and IEEE Smart Data (SmartData).

[21] Fan Kuan, Bao Zijian, Liu Mingxi, Vasilakos Athanasios V., Shi Wenbo (2020). Dredas: decentralized, reliable and efficient remote outsourced data auditing scheme with blockchain smart contract for industrial iot. Future Gener. Comput. Syst..

[22] Ahene Emmanuel, Walker Joojo, Gyening Rose-mary Owusuaa Mensah, Abdul-Salaam Gaddafi, Hayfron-Acquah James Ben (2022). Heterogeneous signcryption with proxy re-encryption and its application in ehr systems. Telecommun. Syst..

[23] Al Baqari Mohammed, Barka Ezedin (2020). 2020 International Wireless Communications and Mobile Computing (IWCMC).

[24] Jabbar Rateb, Fetais Noora, Krichen Moez, Barkaoui Kamel (2020). 2020 IEEE International Conference on Informatics, IoT, and Enabling Technologies (ICIoT).

[25] Sujihelen L. (2023). An efficient chain code for access control in hyper ledger fabric healthcare system. e-Prime-Adv. Electri. Eng. Electron. Energy.

[26] Adlam Ryno, Haskins Bertram (2020). Proceedings of the 2nd International Conference on Intelligent and Innovative Computing Applications.

[27] Chelladurai Usharani, Pandian Seethalakshmi (2022). A novel blockchain based electronic health record automation system for healthcare. J. Ambient Intell. Humaniz. Comput..

[28] Dubovitskaya Alevtina, Baig Furqan, Xu Zhigang, Shukla Rohit, Zambani Pratik Sushil, Swaminathan Arun, Jahangir Md Majid, Chowdhry Khadija, Lachhani Rahul, Idnani Nitesh (2020). Action-ehr: patient-centric blockchain-based electronic health record data management for cancer care. J. Med. Internet Res..

[29] Román-Martínez Isabel, Calvillo-Arbizu Jorge, Mayor-Gallego Vicente J., Madinabeitia-Luque Germán, Estepa-Alonso Antonio J., Estepa-Alonso Rafael M. (2023). Blockchain-based service-oriented architecture for consent management, access control, and auditing. IEEE Access.

[30] Cha Shi-Cho, Meng Weizhi, Li Wen-Wei, Yeh Kuo-Hui (2023). A blockchain-enabled iot auditing management system complying with iso/iec 15408-2. Comput. Ind. Eng..

[31] Lie Martin Forsberg, Sánchez-Gordón Mary, Colomo-Palacios Ricardo (2020). Proceedings of the 14th ACM/IEEE International Symposium on Empirical Software Engineering and Measurement (ESEM).

[32] Aldosari Bakheet (2012). An evaluation of ehr system audit functions in a Saudi Arabian hospital. J. Health Inf. Dev. Ctries..

[33] Wu Danny T.Y., Smart Nikolas, Ciemins Elizabeth L., Lanham Holly J., Lindberg Curt, Zheng Kai (2017). AMIA Annual Symposium Proceedings, vol. 2017.

[34] Rose Christian, Thombley Robert, Noshad Morteza, Lu Yun, Clancy Heather A., Schlessinger David, Li Ron C., Liu Vincent X., Chen Jonathan H., Adler-Milstein Julia (2023). Team is brain: leveraging ehr audit log data for new insights into acute care processes. J. Am. Med. Inform. Assoc..

[35] Sookhak Mehdi, Jabbarpour Mohammad Reza, Safa Nader Sohrabi, Yu F. Richard (2021). Blockchain and smart contract for access control in healthcare: a survey, issues and challenges, and open issues. J. Netw. Comput. Appl..

[36] Tith Dara, Lee Joong-Sun, Suzuki Hiroyuki, Wijesundara W.M.A.B., Taira Naoko, Obi Takashi, Ohyama Nagaaki (2020). Application of blockchain to maintaining patient records in electronic health record for enhanced privacy, scalability, and availability. Healthc. Inform. Res..

[37] Zaabar Bessem, Cheikhrouhou Omar, Jamil Faisal, Ammi Meryem, Abid Mohamed (2021). Healthblock: a secure blockchain-based healthcare data management system. Comput. Netw..

[38] Zheng Zibin, Xie Shaoan, Dai Hong-Ning, Chen Weili, Chen Xiangping, Weng Jian, Imran Muhammad (2020). An overview on smart contracts: challenges, advances and platforms. Future Gener. Comput. Syst..

[39] Mohammed Alaa Hamid, Abdulateef Alaa Amjed, Abdulateef Ihsan Amjad (2021). 2021 3rd International Congress on Human-Computer Interaction, Optimization and Robotic Applications (HORA).

[40] Thatikonda Ramya, Vaddadi Srinivas Aditya, Arnepalli Pandu Ranga Rao, Padthe Adithya (2023). Securing biomedical databases based on fuzzy method through blockchain technology. Soft Comput..

[41] Putra Guntur Dharma, Dedeoglu Volkan, Kanhere Salil S., Jurdak Raja (2020). 2020 IEEE International Conference on Blockchain and Cryptocurrency (ICBC).

[42] Wang Xiaosong, Peng Yifan, Lu Le, Lu Zhiyong, Bagheri Mohammadhadi, Summers Ronald M. (2017). Proceedings of the IEEE Conference on Computer Vision and Pattern Recognition.

